# A Survey on Current Practices of Umbilical Cord Clamping in Malaysia

**DOI:** 10.3389/fmed.2022.917129

**Published:** 2022-07-07

**Authors:** Kwai Meng Pong, Norezliani Puasa, Zaleha Abdullah Mahdy

**Affiliations:** ^1^Paediatric Department, Penang Adventist Hospital, Penang, Malaysia; ^2^Department of Obstetrics and Gynaecology, Faculty of Medicine, Universiti Kebangsaan Malaysia, Kuala Lumpur, Malaysia

**Keywords:** obstetricians, midwives, pediatricians, umbilical cord clamping, practices and knowledge

## Abstract

**Background:**

Delayed cord clamping (DCC) has been demonstrated to have significant benefits in reducing the incidence of intraventricular hemorrhage, blood transfusion and neonatal mortality in preterm neonates and improving hemodynamic and long-term neurodevelopment among term infants. There is no clear guideline on umbilical cord clamping (UCC) practices in Malaysia.

**Objective:**

The aim of this survey was to assess the knowledge and practice of DCC among obstetric doctors and midwives in Malaysia, and pediatric colleagues who witness the delivery.

**Method:**

This is a cross-sectional survey conducted in childbirth facilities in Malaysia from October 2020 to January 2021. A convenient snowball sampling was adopted. A validated questionnaire was disseminated to practicing obstetric and pediatric doctors and midwives electronically via email and WhatsApp using Google Form. The data were analyzed using descriptive and analytical statistics.

**Results:**

A total of 327 respondents completed the questionnaires, comprising 206 obstetric doctors, 72 pediatric doctors and 49 midwives. The majority of respondents were specialists or higher in rank (53.2%). Only 29% reported the existence of guidelines on UCC in their place of work. Midwives (*P* = 0.003) and staff of lower ranks and level of education (*P* < 0.001) appeared to be more aware of the existence of a UCC guideline. Most respondents had positive knowledge of DCC for both term and preterm neonates. A large proportion (82%) of respondents agreed that DCC helped increase neonatal iron stores, and was good for both preterm (70.7%) and term (76.2%) neonates not requiring positive pressure ventilation. Doctors, specialists, those who are 40 years old and above, and those who have been in service for at least 10 years were found to have better knowledge regarding DCC (*P* < 0.05).

**Conclusion:**

The awareness and practice of obstetric, pediatric and midwifery staff of guidelines on UCC were less than satisfactory. Even though most respondents have good knowledge and positive perception regarding benefits of DCC, these were not translated into their routine practice. Hence, a national guideline emphasizing the benefits of DCC should be made available in all childbirth facilities.

## Introduction

Delayed cord clamping (DCC) has been demonstrated to reduce the incidence of intraventricular hemorrhage and the need for neonatal blood transfusion in preterm neonates (1). This practice has also reduced neonatal mortality compared to early clamping in preterm infants (1, 2). The World Health Organization (WHO) recommends DCC >1 min after birth (3) as it can significantly improve hemodynamic and long-term neurodevelopment in term infants (4, 5).

Early or immediate cord clamping (ECC or ICC) is generally defined as clamping of the cord within the first 60 s after birth, and late or DCC is performed more than 60 s after birth or upon cessation of cord pulsation (4, 5).

Although the American College of Obstetricians and Gynecologists (ACOG) recommends a DCC in vigorous term and preterm infants for at least 30–60 s after birth (6), a few studies on DCC practices among healthcare professionals in different countries showed that there are variations in the management of umbilical cord clamping (UCC) and cord milking during normal births (7, 8).

Nevertheless, despite a set time of UCC recommended by WHO and ACOG, some healthcare workers used cessation of cord pulsation or waited until the expulsion of placenta as a set time for UCC (9, 10).

The extent to which this procedure is being practiced in Malaysia is unknown. The aim of this study was to assess the knowledge and practice of DCC among obstetric doctors and midwives in Malaysia, as reported by themselves or by pediatric colleagues who witness the delivery.

## Materials and Methods

We conducted a cross-sectional survey involving healthcare setups with childbirth facilities, including private hospitals and maternity centers, government hospitals and teaching hospitals in Malaysia over a period of 3 months from October 2020 to January 2021.

The inclusion criteria were obstetric doctors and midwives who conducted deliveries, and pediatric doctors who witnessed deliveries. Doctors and midwives who were not actively conducting or witnessing childbirths, e.g., retired professionals, were excluded from this survey.

This study was approved by the Universiti Kebangsaan Malaysia (UKM) Research Ethics Committee (Approval Code: JEP-2020-580).

A validated questionnaire in English on UCC practice and knowledge toward DCC from a published survey (11) was used with permission from the previous researcher. The questionnaire was sent to members of the Perinatal Society of Malaysia (PSM) and the Malaysian Pediatric Association (MPA), as well as to individual practicing obstetric and pediatric doctors and midwives in Malaysia via email or WhatsApp using Google Form. A convenient snowball sampling method was deployed.

The questionnaire covered three domains: (1) demographic data, (2) existing UCC practice, and (3) knowledge about DCC. Consent was obtained from all respondents who were provided with information regarding the survey within the Google Form.

### Sample Size

The sample size was calculated using Statcalc based on a study done by Ibrahim et al. on current UCC practices and knowledge of obstetricians and midwives toward DCC in Saudi Arabia (11). Based on this study, 70% of the respondents have good knowledge toward DCC. By using Statcalc application version 7.2.4.0, with a confidence interval of 95% and margin of error of 5%, we estimated a sample size of 323. Considering 20% dropouts, we aimed to recruit 388 respondents.

### Statistical Analyses

The data were analyzed using the Statistical Package for Social Sciences version 22 (IBM SPSS Statistics 22) for Windows. Results were expressed in percentages. Analyses of comparison of the percentages were conducted using the chi-square test or Fisher's exact test (for non-parametric data). In all analyses, *p* < 0.05 was taken to indicate statistical significance.

## Results

### Study Flow Chart

**Figure 1:**
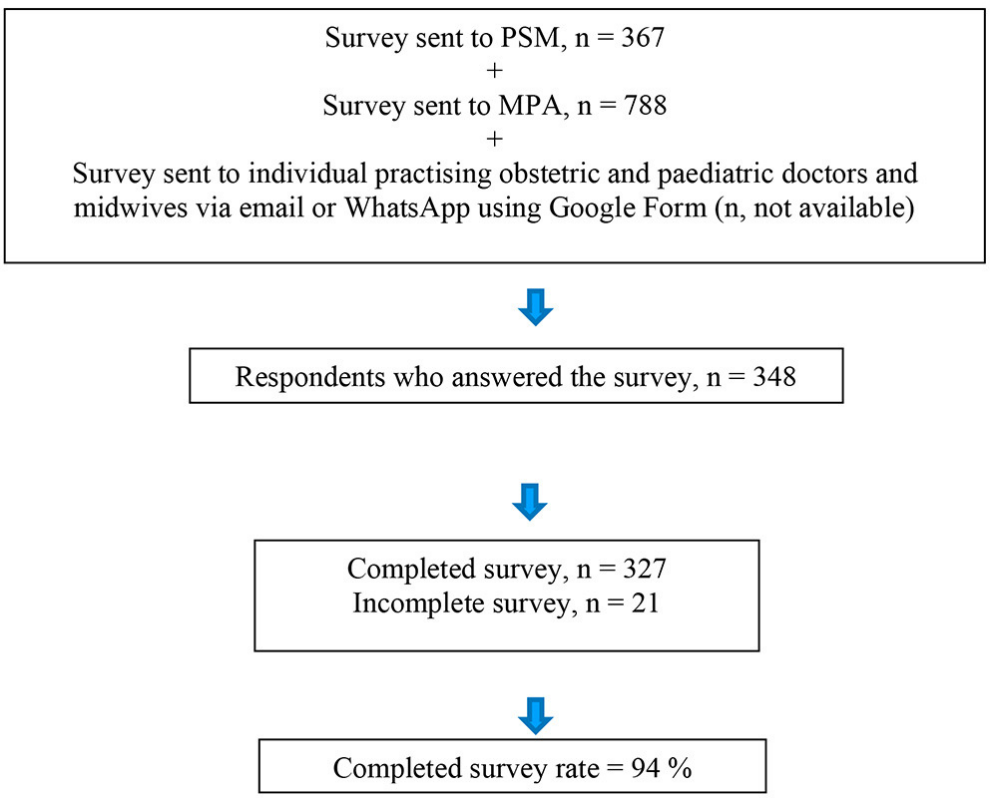
Flow diagram showing the distribution of the survey questionnaire, and the number of respondents who answered and who completed the survey.

The questionnaire was sent to 367 and 788 members of PSM and MPA respectively (Figure 1). It was also disseminated to individual practicing obstetric and pediatric doctors and midwives in Malaysia via email or WhatsApp using Google Form.

Out of a total of 348 respondents who answered the questionnaire, 327 respondents completed it. This number comprised 206 obstetric doctors, 72 pediatric doctors and 49 midwives. Meanwhile, 21 respondents did not complete the questionnaire. The completed survey rate was therefore 94%.

#### Demographic Background of Respondents

The majority of the respondents were females (72%), specialists, fellows or consultants (53%), aged between 30–39 years old (53%), and practicing at Government Hospitals (57%). Most respondents had tertiary educational background (99%) and about a third of the respondents were senior staff with working experience of more than 15 years (33.3%) (Table 1).

**Table 1:** Demographic and characteristics of respondents (*n* = 327).

**Demographic and characteristics**	***n* (%)**
**Profession**	
Obstetric	206 (63.0)
Pediatric	72 (22.0)
Midwife	49 (15.0)
**Level of expertise among doctors**	
Houseman	6 (2.2)
Medical Officer	86 (30.9)
Registrar	38 (13.7)
Specialist	53 (19.0)
Fellow	13 (4.7)
Consultant	82 (29.5)
**Gender**	
Male	90 (27.5)
Female	237 (72.5)
**Age (years old)**	
20–29	22 (6.7)
30–39	172 (52.6)
40–49	70 (21.4)
50–59	54 (16.5)
>60	9 (2.8)
**Place of practice**	
Private hospital/Maternity Center	87 (26.6)
Government hospital (KKM)	187 (57.2)
University/Academic hospital	53 (16.2)
**State of Malaysia where you practice**	
Federal Territory of Kuala Lumpur	73 (22.3)
Federal Territory of Labuan	4 (1.2)
Federal Territory of Putrajaya	49 (15)
Johor	28 (8.6)
Kedah	7 (2.2)
Kelantan	8 (2.4)
Malacca	3 (0.9)
Negeri Sembilan	17 (5.2)
Pahang	8 (2.4)
Perak	15 (4.6)
Perlis	1 (0.3)
Penang	32 (9.8)
Sabah	10 (3.1)
Sarawak	8 (2.4)
Selangor	61 (18.7)
Terengganu	3 (0.9)
**Education level**	
High school	5 (1.5)
Diploma	35 (10.7)
Bachelor	129 (39.5)
Master	153 (46.8)
PhD	5 (1.5)
**Years of service**	
<1 year	7 (2.1)
1–5 years	66 (20.2)
6–10 years	96 (29.4)
11–15 years	49 (15.0)
>15 years	109 (33.3)

*Values are number and percentage*.

#### Existing Umbilical Cord Clamping Practice/Guideline

The majority of respondents (71%) reported no existing UCC guideline in their workplace. Among 29% of respondents who reported the presence of UCC guideline in their practice, only 44 and 50% reported always practicing or practicing most of the time, respectively.

#### Umbilical Cord Clamping for Term Neonates

About a third of the respondents (35%) reported setting UCC time for term neonates. Among those, the majority (62%) reported setting UCC time at 1–3 min. About one third (35%) reported no reason for this routine UCC practice, 29% followed local UCC protocol, 20% clamped the umbilical cord early to prevent polycythemia or hyperbirubinemia, and 15% clamped the cord when the cord pulsations ceased.

Most respondents (77%) applied earlier UCC or immediate cord clamping when the neonates have poor Apgar scores. Other reasons for earlier UCC were increased vaginal blood loss (41%) and presence of nuchal cord (32%). A fifth (20%) of them reported occasions when early UCC took place without any specific reason.

On the other hand, among occasions for later UCC, most of the respondents (40%) reported no specific reason for practicing that. One third (33%) performed later UCC to fulfill the wish of the parents and 16% waited for cessation of umbilical cord pulsation.

#### Umbilical Cord Clamping for Preterm Neonates

In preterm neonates, only 26% of the respondents reported a set UCC time. Half of these respondents (49%) reported a set UCC time of 1–3 min. About a third (35 and 32% respectively) of the respondents reported that DCC benefits or ECC benefits to pediatricians (especially when resuscitation is needed) were important for their routine UCC in preterm neonates.

#### UCC Time in Cesarean Section

During elective cesarean section, 22% of respondents practiced UCC at the same time as vaginal delivery and 20% reported cord milking. Most of them (61%) reported UCC time of 30–59 s. On the other hand, during emergency cesarean section, 20% of the respondents practiced UCC as soon as possible, 17 and 15%, respectively, reported cord milking or practiced UCC at the same time as performed in vaginal delivery. Out of those who reported a specific UCC time during this procedure, 40% carried out UCC at 30–59 s (Table 2).

**Table 2:** Existing umbilical cord clamping practices (*n* = 327).

**Item**	**Options**	**Number of responses, *n***	**%**
Existing guidelines for UCC	Yes	95	29.0
	No	232	71.0
UCC practice following existing guideline (If answer YES)	Always	42	44.2
	Most of the time	48	50.5
	Sometimes	5	5.3
	Never	0	0.0
Set UCC time for term neonate	Yes	116	35.5
	No	211	64.5
Time of UCC in term neonate if answer YES	0–29 s	13	11.2
	30–59 s	28	24.1
	1–3 min	72	62.1
	4–10 min	2	1.7
	Others	1	0.9
Time of UCC in term neonate if answer NO	0–29 s	64	30.3
	30–59 s	85	40.3
	1–1 min 59 s	34	16.1
	2–3 min 59 s	7	3.3
	4–10 min	2	1.0
	>10 min	0	0
	Until pulsations have ceased	14	6.6
	Until the placenta detached	1	0.5
	Other	4	1.9
UCC routine^*^	No reason	114	34.9
	UCC according to a protocol	96	29.4
	UCC to prevent polycythemia or hyperbilirubinemia	64	19.6
	Wait as long as possible; not worried about polycythemia or hyperbilirubinemia	33	10.1
	Wait until the pulsations have ceased to optimize blood supply	50	15.3
	Wait until normal neonatal breathing to optimize blood supply	31	9.5
	Administration of meds (e.g., oxytocin) during AMTSL	16	4.9
	Other	24	7.3
Occasions for earlier UCC ^*^	No reason	67	20.5
	Low APGAR score	252	77.1
	Excessive vaginal blood loss	134	41.0
	Short umbilical cord	54	16.5
	Neonate has Hypothermia	36	11.0
	Nuchal cord	106	32.4
	Wish of the parents	18	5.5
	Pulsations have already ceased	32	9.8
	Placental detachment from the uterine wall	52	15.9
	Administration of oxytocin/other uterotonics	14	4.3
	Low position of the infant	13	4.0
	To prevent polycythemia/hyperbilirubinemia	37	11.3
	Other	8	2.5
Occasions for later UCC^*^	No reason	132	40.4
	Umbilical cord is still pulsating	51	15.6
	Placenta is still attached to the uterine wall	32	9.8
	The mother is breastfeeding	9	2.7
	No vaginal blood loss	67	20.5
	Wish of the parents	109	33.3
	Other	13	4.0
Set UCC time in preterm neonate	Yes	84	25.7
	No	243	74.3
Time of UCC in preterm neonate if answer YES	0–29 s	10	11.9
	30–59 s	30	35.7
	1–3 min	41	48.8
	4–10 min	2	2.4
	>10 min	0	0.0
	Others	1	1.2
Time of UCC in preterm neonate if answer NO	0–29 s	105	43.2
	30–59 s	77	31.7
	1–1 min 59 s	30	12.4
	2–3 min	9	3.7
	4–10 min	1	0.4
	> 10 min	1	0.4
	Until pulsations have ceased	13	5.3
	Until the placenta detached	1	0.4
	Others	6	2.5
Reason of UCC when neonate is preterm	No reason	14	4.3
	ECC benefits are important to pediatricians	106	32.4
	DCC benefits are important	115	35.1
	Condition of baby is important	15	4.6
	Others	77	23.6
UCC time in elective cesarean section	Same time as in vaginal delivery	73	22.3
	As soon as possible	25	7.6
	Cord stripping or milking	64	19.6
	Specific UCC time	44	13.5
	Not applicable (not obstetrician/ O&G trainee)	121	37.0
If have specific UCC time in elective cesarean section	0–29 s	2	4.5
	30–59 s	27	61.4
	1–3 min	6	13.6
	4–10 min	0	0.0
	>10 min	1	2.3
	Other	8	18.2
UCC time in emergency cesarean section	Same time as in vaginal delivery	49	15.0
	As soon as possible	66	20.2
	Cord stripping or milking	56	17.1
	Specific UCC time	35	10.7
	Not applicable (not obstetrician/ O&G trainee)	121	37.0
If have specific UCC time in emergency cesarean section	0–29 s	1	2.9
	30–59 s	14	40.0
	1–3 min	9	25.7
	4–10 min	0	0
	>10 min	0	0
	Other	11	31.4

* *More than one response allowed*.

*ATMSL, active third stage of labor*.

#### Existing UCC Practices With Demographic Characteristic

There was a significantly higher rate of reporting the existence of a UCC guideline among midwives compared to doctors (47 vs. 26%, *P* = 0.003), respondents practicing in government hospital compared to private hospitals (35 vs. 14%, *P* < 0.001) and lower education compared to higher education (60 vs. 25%, *P* < 0.001). Government hospitals were hospitals under the Ministry of Health (MOH) while teaching hospitals were governed under the Ministry of Higher Education (MOHE). Lower education comprised secondary education and diploma, while higher education included bachelor's degree, master and PhD. One factor that was significantly associated with a higher rate of reporting of a set time of UCC for term neonates was lower education (50%) compared to 33% among staff with high level of education (*P* = 0.04). Meanwhile, for preterm neonates, significantly higher rates of reporting of a set time of UCC were found among respondents from Klang Valley (34%), compared to 20% among non-Klang Valley residents (*P* = 0.006). Similar associations were observed among those with lower education (45 vs. 23% in higher, *P* = 0.003). The Klang Valley comprises mainly urban areas in Kuala Lumpur and Selangor in the central west coast of the Malaysian peninsular (Table 3).

**Table 3:** Existing UCC practices with demographic characteristic.

**Characteristics**	**Total number**	**Number with characteristics**	**%**	***P***
**Existence of UCC guidelines/ protocols (*****n** **=*** **95)**				
**Profession**				
Doctor	278	72	25.9	0.003^*^
Midwife	49	23	46.9	
**Level of expertise among doctors (*****n** **=*** **72)**				
Specialist	148	42	28.4	0.314
Non specialist	130	30	23.1	
**Gender**				
Male	90	28	31.1	0.613
Female	237	67	28.3	
**Age**				
<40 years old	194	61	31.4	0.250
≥40 years old	133	34	25.6	
**Place of practice**				
Government hospital	240	83	34.6	<0.001^*^
Private hospital	87	12	13.8	
**State of Malaysia where you practice**				
Klang Valley	134	45	33.6	0.133
Outside Klang Valley	193	50	25.9	
**Education level**				
Low	40	24	60.0	<0.001^*^
High	287	71	24.7	
**Years of service**				
<10 years	169	52	30.8	0.479
≥10 years	158	43	27.2	
**Existence of set time for UCC for term neonates (*****n** **=*** **116)**				
**Profession**				
Doctor	278	97	34.9	0.600
Midwife	49	19	38.8	
**Level of expertise among doctors (*****n** **=*** **97)**				
Specialist	148	55	37.2	0.397
Non-specialist	130	42	32.3	
**Gender**				
Male	90	39	43.3	0.067
Female	237	77	32.5	
**Age**				
<40 years old	194	74	38.1	0.223
≥40 years old	133	42	31.6	
**Place of practice**				
Government hospital	240	88	36.7	0.454
Private hospital	87	28	32.2	
**State of practice in Malaysia**				
Klang Valley	134	49	36.6	0.731
Outside Klang Valley	193	67	34.7	
**Education level**				
Low	40	20	50.0	0.040^*^
High	287	96	33.4	
**Years of service**				
<10 years	169	60	35.5	0.991
≥10 years	158	56	35.4	
**Existence of set time for UCC for preterm neonates (*****n** **=*** **84)**				
**Profession**				
Doctor	278	69	24.8	0.392
Midwife	49	15	30.6	
**Level of expertise among doctors (*****n** **=*** **69)**				
Specialist	148	37	25.0	0.941
Non-specialist	130	32	24.6	
**Gender**				
Male	90	28	31.1	0.167
Female	237	56	23.6	
**Age**				
<40 years old	194	48	24.7	0.636
≥40 years old	133	36	27.1	
**Place of practice**				
Government hospital	240	11	4.6	0.350
Private hospital	87	2	2.3	
**State of Malaysia where you practice**				
Klang Valley	134	45	33.6	0.006^*^
Outside Klang valley	193	39	20.2	
**Education level**				
Low	40	18	45.0	0.003^*^
High	287	66	23.0	
**Years of service**				
<10 years	169	42	24.8	0.720
≥10 years	158	42	26.6	

*Data presented as n (%) and analyzed by Chi-square test*.

*Specialist includes fellow and consultant. Non-specialist consists of houseman, medical officer and registrar*.

*Government hospital includes all hospitals under MOH and MOE*.

*Low education consists of high school and diploma. High education includes bachelor, master and PhD*.

*Klang valley consists of areas in Kuala Lumpur and Selangor*.

* *Statistically significant difference*.

#### Knowledge on Delayed Cord Clamping

More than two thirds (71% and 76%) of the respondents agreed that DCC was good for both preterm and term neonates not requiring PPV, respectively. The majority of the respondents (82%) agreed that DCC increased iron stores during the neonatal period in both preterm and term babies. Nearly three quarters (73%) respondents agreed that DCC has valuable effects that extend beyond the neonatal period including better long-term neurodevelopment while 67% agreed that DCC helped stabilize the transition of circulation, lessening the need for inotropic medications and reducing blood transfusion, necrotizing enterocolitis and intraventricular hemorrhage in preterm babies (Table 4).

**Table 4:** Attitude and knowledge toward delayed cord clamping (Likert items).

**Item**	**Mean**	**SD**	**Percentage agree/strongly agree**
DCC is good for preterm neonates who do not require PPV	3.82	0.88	70.7
DCC is good for term neonates who do not require PPV.	3.94	0.86	76.2
DCC can help to increase iron stores in term and preterm babies.	3.98	0.66	82.0
DCC has valuable effects that extend beyond the neonatal period by increasing the iron stores, which include improvements in long term neurodevelopment.	3.84	0.70	72.8
DCC helps in stabilizing the transition of circulation, lessening the need for inotropic medications and reducing blood transfusions, necrotizing enterocolitis and intraventricular hemorrhage in preterm babies.	3.80	0.71	67.3

*Coding: 1 = strong disagree, 2 = disagree, 3 = not sure, 4 = agree, 5 = strongly agree*.

Among the respondents, doctors, specialists, males, those who are 40 years old and above, those who have been in service for at least 10 years were found to have better knowledge regarding DCC (*P* < 0.05) (Table 5).

**Table 5:** Number and percentage of respondents with positive attitude and good knowledge toward DCC by demographic characteristics.

**Characteristic**	**Total number**	**Number with good knowledge (*n =* 151)**	**%**	***P***
**Profession**				
Doctor	278	137	49.3	0.007^*^
Midwife	49	14	28.6	
**Level of expertise among doctors (*****n** **=*** **137)**				
Specialist	148	90	60.8	<0.001^*^
Non-specialist	130	47	36.2	
**Gender**				
Male	90	53	58.9	0.004^*^
Female	237	98	41.4	
**Age**				
<40 years old	194	80	41.2	0.030^*^
≥40 years old	133	71	53.4	
**Place of practice**				
Government hospital	187	80	42.8	0.154
Private hospital	140	71	50.7	
**State in Malaysia where you practice**				
Klang Valley	134	60	44.8	0.672
Outside Klang valley	193	91	47.2	
**Education level**				
Low	40	16	40.0	0.403
High	287	135	47.0	
**Years of service**				
<10 years	169	68	40.2	0.026^*^
≥10 years	158	83	52.5	

*Positive attitude and good knowledge is defined by selecting strongly agree and agree for all 5 statements about DCC as shown in Table 4. Data presented as n (%) and analyzed by Chi-square test*.

* *Statistically significant difference*.

## Discussion

Our study revealed the state of practice of UCC and level of knowledge regarding DCC among obstetric and pediatric doctors, and midwives in Malaysia. The majority (71%) of respondents were not aware of any formal guideline on UCC in their place of practice. Similar findings were seen in previous studies by Ibrahim et al. (11) and Jelin et al. (12). Despite published evidence on the benefits associated with DCC (1–3, 6, 13, 14), only 29% of respondents reported that their institution have a UCC guideline. This was contrary to obstetric practice in a developed country, where DCC is considered a standard of care (6, 13).

One statistically significant finding in reporting the existence of a UCC guideline in the workplace was the significant disparity between midwives (47%) and doctors (26%) (*P* = 0.003). This was contrary to the observation in the study by Ibrahim et al. (11) where doctors were more aware of the availability of a UCC guideline compared to midwives.

Another survey conducted among midwives in Irish hospitals (14) reported 64.7% of the midwives were aware of the guideline on DCC in their place of practice. Our study also found a statistically significant difference in the reporting of the existence of a set time for UCC for term neonates between staff with lower education vs. those with higher education (60 vs. 25%, *P* = < 0.001 and 50 vs. 33%, *P* = 0.04, respectively). Lower education included high school and diploma, indicating midwives. These findings were consistent with the findings of studies by Boerre et al. (9) and Mercer et al. (15). Furthermore, some midwifery institutions such as the American College of Nurse-Midwives (ACNM) have adopted DCC as a standard practice for both preterm and term newborns (16).

Midwives have slightly higher rates of having a set time for UCC for both term and preterm neonates than doctors (39 vs. 35% and 31 vs. 25%, respectively), although the differences were not statistically significant (*P* = 0.600 and 0.392 respectively). Similar findings were seen in a study by Boerre et al. (9). Interestingly, the UCC practice among midwives was said to be based more on belief, experience and clinical training rather than scientific rationale (15). This possibly explains the disparity between knowledge and practice in our survey, with better DCC practice among midwives, despite poorer knowledge. At the level of diploma education, the emphasis is on technicalities and practice, rather than deep knowledge and scientific evidence. This is the level of education of the vast majority of midwives in Malaysia.

DCC for at least 30–60 s for vigorous term and preterm newborns who do not require resuscitation at birth was incorporated into the curriculum of Neonatal Resuscitation Program (NRP) 7th edition (17). In fact, umbilical cord management plan has been included as one of the 4 important pre-birth questions to the obstetric provider in the NRP 8th edition which was launched in 2021 (18). One of the possible explanation for the above observation in the UCC in Malaysia is that NRP is a mandatory course funded by the government for all the pediatric and obstetrics house officers, medical officers and midwives in Malaysian public and teaching hospitals (19). These categories of providers had the opportunities to learn about the latest information of the DCC guidelines in their NRP training compared to their more senior colleagues, e.g., the fellows and the consultants as well as their counterparts in the private sectors.

A systematic review demonstrated that there were no significant differences between ECC and DCC with regard to adverse neonatal outcomes such as low Apgar score and admission to special care nursery (SCN) or neonatal intensive care unit (NICU), and adverse maternal outcomes such as severe postpartum hemorrhage (4). Having said that, majority of the respondents (77% and 41%) applied ECC to neonates with poor Apgar scores and where mothers had increased vaginal loss, respectively, in this study. Such practices were usually performed in order to assist in neonatal resuscitation or to prevent postpartum hemorrhage (20).

Among the respondents who reported a set UCC time, (26% in preterm and 35% in term neonates), most of them reported a set UCC time of 1–3 min (49% in preterm and 62% in term). This practice is consistent with the recommendation by WHO (3), whereby in both preterm and term neonates who do not require PPV, the cord should not be clamped earlier than 1 min after birth. DCC (no earlier than 1 min after birth) is recommended to improve both maternal and infant health and nutritional outcomes.

The majority of respondents (71 and 76%, respectively) agreed that DCC was good for preterm and term neonates not requiring PPV. These findings are consistent with the study done by Ibrahim et al. (11) where the majority of respondents agreed that DCC is beneficial for both term (69%) and preterm (71%) neonates not requiring PPV. In fact, DCC in preterm neonates who require PPV can now be facilitated by providing a mobile resuscitation trolley before UCC (21) and this was proven to be feasible as demonstrated by Lapcharoensap et al. (22) and Joshi et al. (23). Having said that, only 30% of the respondents agreed that DCC was good for neonates who require PPV. Similar findings were reported by Leslie et al. (13). This may be due to its safety concern and acceptability to clinician and parents of DCC in a newborn who requires PPV is still being investigated (21).

Like previous studies (9, 11), majority of the respondents (82%) agreed that DCC increases iron stores during the neonatal period in both preterm and term babies. Most (73%) respondents agreed that DCC has valuable effects that extend beyond the neonatal period, including better long term neurodevelopment. Two thirds (67%) agreed that DCC helped stabilize the transition of circulation, lessening the need for inotropic medications and reducing blood transfusion, necrotizing enterocolitis and intraventricular hemorrhage in preterm babies. These findings are consistent with a previous systematic review and meta-analyses, which supports current guidelines recommending DCC in preterm infants (4, 24).

In our study, doctors, specialists, males, those who are 40 years old and above, and those who have been in service for at least 10 years have better knowledge regarding DCC. This is quite similar to the findings in the study by Ibrahim et al. (11), where males and those with more than 16 years of practice in obstetrics had good knowledge of DCC. The relatively lower level of knowledge regarding delayed cord clamping among midwives and non-specialists is a matter of concern, because in Malaysia the majority of deliveries in public facilities are conducted by midwives and medical officers who are not specialists yet, and calls for more efforts to educate these two groups of healthcare workers on this issue. The older age and seniority are probably confounded by the specialist status of those who scored well on knowledge assessment.

The strength of this study was the high completed survey rate of 94%. Besides, this study utilized a widely-used validated questionnaire to measure the practice of UCC and knowledge regarding DCC. Furthermore, this was the first study that assessed obstetric and pediatric doctors and midwives on UCC practices and knowledge regarding DCC in Malaysia. It is also the first such survey in the South East Asia region. We hope to alert not only our fellow Malaysian healthcare workers in the related fields, but also regional healthcare professionals, especially in our neighboring developing countries. Similarly, we hope to raise awareness in other Low to Moderate Income Countries (LMICs) elsewhere in the world.

One limitation of this study was that the participants were selected by convenient snowball sampling through email and WhatsApp using Google form, hence not all hospitals in Malaysia participated. We were unable to capture the actual numbers of invitation sent to the potential respondents because of the overlap in membership among the three professional societies, namely the Perinatal Society of Malaysia (PSM), the Malaysian Paediatric Association (MPA), and the Obstetrical and Gynaecological Society of Malaysia (OGSM). Although we were able to send the invitation to more than 1,000 members from PSM (*n* = 367) and MPA (*n* = 788) however only about one third (*n* = 348) of them returned the survey form.

## Conclusion

Awareness of the existence of guidelines on UCC was still markedly lacking among Malaysian professionals handling childbirth. Even though most respondents had good knowledge of DCC, this did not translate into their routine practice. Hence, a national guideline emphasizing the benefits of DCC should be made available in Malaysia. This may also help create awareness of the existence of international UCC guidelines.

## Data Availability Statement

The original contributions presented in the study are included in the article/Supplementary Material, further inquiries can be directed to the corresponding author.

## Ethics Statement

The studies involving human participants were reviewed and approved by Universiti Kebangsaan Malaysia (UKM) Research Ethics Committee (Approval Code: JEP-2020-580). The patients/participants provided their written informed consent to participate in this study.

## Author Contributions

Conceptualization by KP who steered the project and critically edited the manuscript. NP prepared the first draft of this manuscript, created the survey, analyzed and collated the data, statistics from the survey, contributed in the writing, created the table and figure, accessed the statistics from sources, ensured editorial accuracy of the manuscript, and reference compilation. ZM critically edited, primarily involved in re-structuring, and re-writing and re-formatting the manuscript into the final form. All authors contributed to the article and approved the submitted version.

## Conflict of Interest

The authors declare that the research was conducted in the absence of any commercial or financial relationships that could be construed as a potential conflict of interest.

## Publisher's Note

All claims expressed in this article are solely those of the authors and do not necessarily represent those of their affiliated organizations, or those of the publisher, the editors and the reviewers. Any product that may be evaluated in this article, or claim that may be made by its manufacturer, is not guaranteed or endorsed by the publisher.
