# Supplementary material for: A Survey on Current Practices of Umbilical Cord Clamping in Malaysia
Source: Front Med (Lausanne). 2022 Jul 7;9:917129. doi: 10.3389/fmed.2022.917129 (PMC9300882; doi:10.3389/fmed.2022.917129)
Supplement: Supplementary file 1 [file Data_Sheet_1.docx]

**APPENDIX:**

**Survey Form**

**Current practice of umbilical cord clamping in Malaysia: a questionnaire study.**

The purpose of this survey is to assess the practice of delayed cord clamping (DCC) among delivery room teams in Malaysia. This is a questionnaire-based survey conducted by the Penang Adventist Hospital, in collaboration with Universiti Kebangsaan Malaysia. You are invited to participate in this survey because you are listed as a member of the Perinatal Society of Malaysia (PSM), the Malaysian Paediatric Association (MPA), or the Obstetrical and Gynaecological Society of Malaysia (OGSM).

*Doctors and midwives who are not actively conducting or witnessing childbirths, e.g. retired professionals,* *are excluded from this study.*

Your participation in this survey is voluntary. You may choose not to participate. If you decide to participate in this survey, you may withdraw at any time. If you decide not to participate in this survey or if you withdraw from participating at any time, you will not be penalized.

This will take approximately 10 minutes. Your responses will be confidential and we do not collect identifying information such as your name, email address or IP address.

If you have any questions about the survey, please contact: Dr Norezliani binti Puasa at [norezliani@gmail.com](mailto:norezliani@gmail.com) or Dr Pong Kwai Meng at [pongkwaimeng@pah.com.my](mailto:pongkwaimeng@pah.com.my) or Professor Dr Zaleha Abdullah Mahdy at [zaleha@ppukm.ukm.edu.my](mailto:zaleha@ppukm.ukm.edu.my).

Your feedback is important. Thank you for participating in our survey.

**Consent for Participation**

I have read and understood the above information, and I agree to participate in this survey.

Yes €

No €Top of Form

Note:

“Yes” will bring you to the questionnaire.

“No” will terminate this Google Form.

Thank you.

**Questionnaire**

A questionnaire from a study entitled **“Current umbilical cord clamping practices and attitudes of obstetricians and midwives toward delayed cord clamping in Saudi Arabia”** obtained after getting an official consent from the author.

**Please select the appropriate answer for each of the following:**

1. Profession:

- Obstetrics
- Paediatrics
- Midwife

If you are a doctor in Obstetrics or Paediatrics, which of these best describes your current level of clinical expertise?

- Houseman
- Medical Officer
- Registrar
- Specialist
- Fellow (subspecialty trainee)
- Consultant

1. Gender:

- Male
- Female

1. Age in years:

- 20- 29
- 30- 39
- 40- 49
- 50- 59
- More than 60

1. Place of practice:

- Private hospital/Maternity Centre
- Government hospital
- University/Academic hospital

1. State of Malaysia where you practice:

- [Federal Territory](https://simple.wikipedia.org/w/index.php?title=Federal_Territory_(Malaysia)&action=edit&redlink=1) of [Kuala Lumpur](https://simple.wikipedia.org/wiki/Kuala_Lumpur)
- [Federal Territory](https://simple.wikipedia.org/w/index.php?title=Federal_Territory_(Malaysia)&action=edit&redlink=1) of [Labuan](https://simple.wikipedia.org/wiki/Labuan)
- [Federal Territory](https://simple.wikipedia.org/w/index.php?title=Federal_Territory_(Malaysia)&action=edit&redlink=1) of [Putrajaya](https://simple.wikipedia.org/wiki/Putrajaya)
- Johor
- Kedah
- Kelantan
- Malacca
- Negeri Sembilan
- Pahang
- Perak
- Perlis
- Penang
- Sabah
- Sarawak
- Selangor
- Terengganu

1. Education level:

- High school
- Bachelor
- Diploma
- Masters
- Doctor (MD)
- PhD

1. How long have you been in practice?

- Less than 1 year
- 1 -5 years
- 6-10 years
- 11-15 years
- More than 15 years

1. Are there any guidelines/protocols for the time of cord clamping in the department you work at?

- No
- Yes
- If Yes, do you follow the guidelines?
- Always
- Most of the time
- Sometimes
- Never

1. Do you have a set cord clamping time when clamping the cord if the neonate is term and healthy with a good APGAR score?

- Yes, go to question 9a then continue till the end of the questionnaire
- No, go to question 9b then continue till the end of the questionnaire

Question 9a: If yes, please report when? Fill in: ………... seconds

Question 9b: If you do not have a set cord clamping time, when do you clamp the cord if the neonate is term and healthy with a good APGAR score?

- Direct 0-29 seconds after birth
- 30 seconds-59 sec after birth
- 1-1 minutes 59 sec after birth
- 2-3 minutes after birth
- 4 – 10 minutes after birth
- > 10 minutes after birth
- I wait until the pulsations have ceased in the umbilical cord.
- I wait until the placenta comes loose from the uterine wall.
- Other.................................................................................................

1. Which statement is applicable for your cord clamping routine? (It is possible to apply more than one answer):

- I do not have a specific reason for my cord clamping routine. This is the way I was taught to do it. I always do it like this.
- I clamp the umbilical cord according to a protocol within my practice/department.
- I clamp the umbilical cord to prevent polycythaemia and hyperbilirubinemia.
- I wait as long as possible before clamping the umbilical cord; I am not worried about polycythaemia and hyperbilirubinemia.
- I find it important to wait until the pulsations have ceased in the umbilical cord to optimize the blood supply to the neonate.
- I find it important to wait until normal neonatal breathing starts so as to optimize the oxygen and blood supply to the neonate
- For me the administration of medication (e.g. oxytocin during active management of third stage of labour) is a reason to clamp the cord.
- Other…………………………………………………………………….

1. On which occasions would you clamp the cord **earlier** than you are used to? (It is possible to apply more than one answer)

- Not applicable. I always clamp the cord immediately
- The neonate has a poor APGAR score
- A lot of vaginal blood loss
- A short umbilical cord
- Hypothermia (low temperature) of the neonate.
- Nuchal cord/ cord round neck (cord clamping done at vulva)
- Parents’ wish
- When the pulsations have ceased.
- When the placental has detach from the uterine wall or has already been delivered.
- The administration of oxytocin or other uterotonics
- Low position of the delivery of the baby (e.g. birth on a birthing stool)
- To prevent polycythaemia/hyperbilirubinemia
- Other…………………………………………….……………………………..

1. In which occasions would you clamp the cord **later** than you are used to? (It is possible to apply more than one answer)

- Parents’ wish
- The umbilical cord is still pulsating at the time when I usually clamp the umbilical cord
- The placenta is still attached to the uterine wall
- The mother is breastfeeding
- No vaginal blood loss
- Not applicable
- Other………………………….………………………….…………………….

1. Do you have a set cord clamping time during a vaginal delivery if the neonate is preterm?

- Yes, go to question 13 a.
- No, go to question 13 b.

Question 13 a: If yes, please report when? Fill in: ………. Seconds

Question 13 b: If you do not have a set cord clamping time when do you clamp the cord if the neonate is preterm?

- Direct 0-29 seconds after birth
- 30 seconds-59 sec after birth
- 1-1 minute 59 sec after birth
- 2-3 minutes after birth
- 4 – 10 minutes after birth
- > 10 minutes after birth
- I wait until the pulsations have ceased in the umbilical cord.
- I wait until the placenta comes loose from the uterine wall.
- Other...................................................................................................................

1. What is the reason for clamping the cord at this time when the neonate is preterm?

.............................................................................................................................................................................................................................................................................................................................................................................................................................................

Questions 15 and 16 are to be answered if you are an obstetrician / O&G trainee.

1. What cord clamping time do you use during an elective caesarean section?

- I clamp the cord at the same time as during a vaginal delivery.
- I clamp the cord as soon as possible.
- Cord stripping or milking.
- I delay clamping by ____ seconds.

1. What cord clamping time do you use during an emergency caesarean section?

- I clamp the cord at the same time as during a vaginal delivery.
- I clamp the cord as soon as possible.
- Cord stripping or milking.
- I delay clamping by ____ seconds.

**Please select the most appropriate response for each of the following regarding delayed cord clamping (DCC) done 1-3 minutes after birth:**

**Please consider:**

1. **Strongly Disagree (2) Disagree (3) Not sure (4) Agree (5) Strongly Agree.**

|  | **(1)** | **(2)** | **(3)** | **(4)** | **(5)** |
| --- | --- | --- | --- | --- | --- |
| 1. DCC is good for preterm babies who do not require positive pressure ventilation. |  |  |  |  |  |
| 1. DCC is good for term babies who do not require positive pressure ventilation. |  |  |  |  |  |
| 1. DCC can help to increase iron stores in term and preterm babies. |  |  |  |  |  |
| 1. DCC has valuable effects that extend beyond the neonatal period by increasing iron stores, which include improvements in long term neurodevelopment. |  |  |  |  |  |
| 1. DCC helps in stabilizing the transition of circulation, lessening the need for inotropic medications and reducing blood transfusions, necrotizing enterocolitis and intraventricular haemorrhage in preterm babies. |  |  |  |  |  |

**Thank you for answering this questionnaire!**
